# Impaired Glucose Tolerance and Altered Body Composition in Obese Young Adults: A Case–Control Study

**DOI:** 10.3390/biomedicines13071569

**Published:** 2025-06-26

**Authors:** Himan Mohamed-Mohamed, Teresa Pardo-Moreno, Margarita Jimenez-Palomares, Bibiana Perez-Ardanaz, Encarnación M. Sánchez-Lara, Maria D. Vazquez-Lara, Mario de La Mata-Fernandez, Victoria García-Morales, Juan José Ramos-Rodríguez

**Affiliations:** 1 Department of Physiology, University of Granada, 18071 Granada, Spain; imanceuta@gmail.com (H.M.-M.); mrdelamata@go.ugr.es (M.d.L.M.-F.); 2 Grupo de Investigación Neurofisiología del Envejecimiento, Faculty of Health Sciences, University of Granada, 18071 Granada, Spain; tpardo@ugr.es (T.P.-M.); victoria.garcia@gm.uca.es (V.G.-M.); 3 Department of Nursing, Faculty of Health Sciences, University of Granada, 18071 Granada, Spain; 4 Department of Biomedicine, Biotechnology and Public Health, University of Cádiz, 11001 Cadiz, Spain; margarita.jimenezpalomares@gm.uca.es; 5 Department of Personality, Faculty of Health Sciences, Assessment and Psychological Treatment, University of Granada, 18071 Granada, Spain; emsanchez@ugr.es; 6 Andalusian Health Service, Department of Nursing, Menendez Tolosa Health Center, 11202 Algeciras, Spain; lolivazquezsas@gmail.com

**Keywords:** body mass index, diabetes, glucose intolerance, insulin resistance, obesity

## Abstract

**Background/Objectives**: To examine the association between body composition and glucose tolerance in young adults with normal weight, overweight, or obesity. **Methods**: This observational case–control study included 154 healthy individuals aged 18–25 years. Participants were categorized into three BMI-based groups and underwent anthropometric and body composition assessments using bioelectrical impedance. Glucose tolerance was evaluated via oral glucose tolerance testing, with capillary blood samples collected at baseline and at 30, 60, 90, and 120 min post load. **Results**: Compared to the normal-weight group, overweight and obese individuals exhibited significantly higher body weight, BMI, visceral and total fat percentages, and reduced muscle mass. Obese participants also showed a significantly greater glucose area under the curve (AUC) and higher fasting and post-load glucose levels. Visceral fat was positively correlated with metabolic impairment. These results indicate a progressive decline in glucose tolerance associated with increasing adiposity and reduced lean mass. **Conclusions**: Young adults with elevated BMI already demonstrate marked alterations in body composition and impaired glucose tolerance, even in the absence of overt metabolic disease. These findings underscore the importance of the early identification of at-risk individuals using simple, non-invasive tools. Preventive strategies promoting healthy body composition in early adulthood may reduce the future risk of diabetes and its associated complications.

## 1. Introduction

Type 2 diabetes mellitus (T2D) has become a major global health concern, with incidence rates increasing markedly across all age groups over the past 25 years. Current data estimate that nearly 10% of adults aged 20–80 years are affected worldwide, with projections indicating that approximately 784 million individuals will be living with T2D by 2045 [1]. T2D constitutes over 90% of all diabetes diagnoses and is defined by progressive insulin resistance and impaired glucose metabolism [2]. Previously regarded as a condition of later adulthood, its onset has now shifted toward younger populations, with the incidence in individuals under 40 tripling in the past 20 years [3]. This trend is attributed to a combination of risk factors, including genetic susceptibility, sedentary behavior, excessive caloric intake, and—most notably—obesity. Comorbidities such as hypertension, dyslipidemia, non-alcoholic fatty liver disease, and albuminuria are frequently observed in younger individuals with T2D [4,5].

A family history remains a strong predictor, with 60% of affected youth reporting at least one parent with T2D and up to 100% indicating a first- or second-degree relative diagnosed with the disease [5,6]. However, excess adiposity—especially central and visceral fat—is broadly recognized as the leading modifiable contributor to early-onset T2D [7]. Notably, nearly 95% of young individuals diagnosed with T2D present with obesity [5].

Obesity triggers a cascade of metabolic disturbances, beginning with insulin resistance and compensatory hyperinsulinemia [2]. The sustained overstimulation of pancreatic β-cells eventually results in functional deterioration and diminished insulin secretion [2,8]. Evidence indicates that this decline occurs more rapidly in younger individuals, with β-cell function decreasing by up to 35% per year, compared to only 7% in those diagnosed after age 40 [8].

The clinical implications of early-onset T2D are considerable as the condition follows a more aggressive progression. Prolonged exposure to hyperglycemia increases the overall disease burden and hastens the onset of microvascular and macrovascular complications [5,9,10]. In this setting, persistent insulin resistance, dyslipidemia, and visceral adiposity emerge as major contributors to elevated cardiovascular risk [11,12]. Liver-related conditions, including non-alcoholic fatty liver disease and its potential evolution into fibrosis or cirrhosis, are also frequently observed [13].

Emerging evidence robustly links excess body weight and glucose intolerance in young adults with insulin resistance, a pivotal factor in the development and progression of T2D. These associations highlight a critical window in youth and early adulthood when metabolic dysfunction begins to accelerate, and targeted interventions could prevent irreversible damage. Research consistently shows that even young individuals with normal glucose levels but increased adiposity or insulin resistance exhibit elevated risks of progressing to prediabetes and T2D, especially in the presence of visceral adiposity or impaired glucose metabolism [14,15,16].

Given these trends, understanding early metabolic alterations in young adults becomes increasingly important. Individuals with excess adiposity may already exhibit subtle disruptions in glucose regulation and body composition, potentially indicating elevated long-term risk. Recent global projections underscore a worrying rise in obesity rates among youth, with prevalence expected to continue increasing through 2050 [17].

This study aimed to explore whether overweight and obesity in young adults are associated with early signs of glucose intolerance and insulin resistance, even in the absence of overt T2D. Based on previous literature, we hypothesized that (1) individuals with higher BMI would exhibit greater insulin resistance and poorer glucose tolerance profiles, (2) women and men may show distinct patterns of association between adiposity and glucose metabolism, and (3) altered body composition, especially increased visceral fat, would be associated with subclinical metabolic disturbances.

## 2. Materials and Methods

### 2.1. Participants

This was a cross-sectional observational study aimed at evaluating glucose tolerance in young adults, stratified by body mass index (BMI). Data were collected between March 2020 and March 2023. The study sample comprised 96 first-year nursing students (25 males and 71 females), aged 18 to 25 years, enrolled at the University of Granada (Spain). Although the sex distribution was unequal, the sample was analyzed as a whole, and sex-specific analyses were not conducted due to limited subgroup size.

The study was approved by the Provincial Research Ethics Committee of Granada (29 March 2022; meeting minutes 3/22). All participants were in good health, had no diagnosed medical conditions, and presented normal fasting plasma glucose levels (80–120 mg/dL) prior to testing.

Inclusion criteria included the following: age between 18 and 25 years, no prior diagnosis of diabetes mellitus, BMI classification according to WHO, and general good health, with no acute or chronic illness at the time of study. Exclusion criteria included the following: pregnancy; use of corticosteroids, hormonal contraceptives, or hormonal therapy; any known endocrine disorders (including polycystic ovary syndrome or thyroid dysfunction); or other chronic illnesses (e.g., renal failure, autoimmune diseases).

Written informed consent was obtained from all individuals following a comprehensive explanation of the study’s aims, procedures, risks, and potential benefits. Participants were informed of their right to withdraw at any time.

BMI was calculated as weight in kilograms divided by squared (m^2^), and participants were categorized into three BMI-based groups [3]:

- Normal weight (BMI 18.5–24.9 kg/m^2^);
- Overweight (BMI 25.0–29.9 kg/m^2^);
- Obesity (BMI ≥30.0 kg/m^2^).

### 2.2. Anthropometric and Body Composition Assessment

Anthropometric measurements were obtained under standardized conditions. Body weight and height were recorded, followed by assessment of muscle mass percentage, body fat percentage, visceral fat, and basal metabolic rate.

Body composition was assessed using the OMRON BF511 (OMRON Healthcare Europe, Hoofddorp, The Netherlands) bioelectrical impedance device, which provides estimates of body fat, visceral fat, muscle mass, and basal metabolic rate. This model has been validated in young adult populations and has shown acceptable agreement with dual-energy X-ray absorptiometry (DEXA) and magnetic resonance imaging (MRI) for estimating visceral fat levels [18,19]. Its non-invasive nature, accessibility, and ease of use make it a suitable tool for field studies and large-scale population screening, as we have previously employed [20].

### 2.3. Glucose Tolerance Testing Young Adults

To evaluate glucose regulation in vivo, participants underwent an oral glucose tolerance test (OGTT) [21] following a two-hour fasting period. A 75 g glucose solution was administered orally, and capillary blood glucose levels were measured at five time points: at baseline (0 min) and at 30, 60, 90, and 120 min post ingestion.

The area under the curve (AUC) was calculated using the trapezoidal method based on capillary glucose values obtained at each time point (0, 30, 60, 90, and 120 min). The AUC was computed as the total (absolute) area under the glucose curve, without subtracting the baseline (0 min) glucose value. Therefore, the values reported correspond to the total AUC, and not to a net or positive incremental AUC, as defined in the literature [21,22].

Given the exploratory and non-diagnostic nature of this study, and the inclusion of healthy young adults, advanced clinical tests such as HbA1c and insulin tolerance tests (ITT) were not included. The metabolic assessment was limited to fasting glucose and OGTT, which are appropriate for identifying early metabolic alterations in this population.

### 2.4. Statistical Analysis

All data were analyzed using SPSS version 23.0 (IBM Corp., Armonk, NY, USA). The normality of continuous variables was assessed using the Shapiro–Wilk test, and the homogeneity of variances was verified using Levene’s test. These assumptions were confirmed prior to performing one-way ANOVA and two-way repeated-measures ANOVA. For glucose tolerance data, sphericity was tested using Mauchly’s test, and the Greenhouse–Geisser correction was applied when the assumption of sphericity was violated. Post hoc comparisons were conducted using Tukey’s b test. A significance level of *p* < 0.05 was considered statistically significant. Data are shown as means ± standard errors of the mean (SEMs).

Due to the limited sample size of the obese group, results should be interpreted with caution. Sex-stratified analysis was not feasible due to unequal distribution.

## 3. Results

### 3.1. Body Height, Weight, and BMI

Anthropometric parameters, including body height, weight, and BMI, were assessed using bioelectrical impedance. Group sizes ranged from sixty-four to seven participants, as shown in Table 1. Statistical differences were evaluated using one-way ANOVA followed by Tukey’s b post hoc test, which was used to evaluate intergroup differences. The data indicated that 66.6% of participants had a normal BMI; 26% were classified as overweight (OW) and 7.3% as obese (OB).

No statistically significant differences were found in height across groups (F(2, 93) = 0.99, *p* = 0.3769). The mean height in the NW group was 1.67 m (CI 95% (1.65 to 1.68)); the OW mean was 1.65 m (CI 95% (1.62 to 1.69)); the OB mean was 1.63 m (CI 95% (1.55 to 1.7)). However, both body weight and BMI values were significantly higher in the OW and OB groups compared with the normal-weight (NW) group. The mean weight in the NW group was 60.92 kg (CI 95% (58.84 to 63.00)); the OW mean was 71.56 (CI 95% (1.64 to 1.69)); the OB mean was 86.57 kg (CI 95% (75.4 to 97.74)). The mean BMI in the NW group was 21.76 (CI 95% (21.34 to 22.18)); the OW mean was 26.09 (CI 95% (25.67 to 26.5)); the OB mean was 32.59 (CI 95% (29.28 to 35.9)). Weight: F(2, 93) = 36.851; BMI: F(2, 93) = 157.225; *p* < 0.001 for both comparisons; see Table 1.

### 3.2. Body Composition

**Figure 1:**
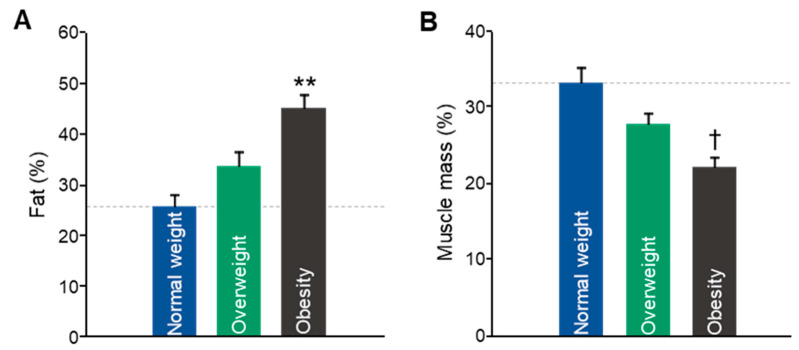
Body fat percentage (**A**) and muscle mass percentage (**B**) in young adults. Data are shown as means ± SEMs. NW (n = 64); OW (n = 25); OB (n = 7). Statistical differences were detected by one-way ANOVA for independent measures followed by Tukey’s b post hoc test (** *p* < 0.05 vs. rest of the groups; † *p* < 0.05 vs. NW group).

Body composition analysis revealed significant differences across BMI groups. Body fat percentage was markedly elevated in the obese (OB) group compared to both normal-weight (NW) and overweight (OW) participants. The mean fat percentage in the NW group was 25.64% (CI 95% (20.68 to 30.62)); the OW mean was 33.74% (CI 95% (27.37 to 40.1)); the OB mean was 45.1% (CI 95% (37.88 to 52.32)). F(2, 28) = 8.119, and *p* = 0.002, as shown in Figure 1A. Although the OW group exhibited a slight increase in fat percentage, the difference was not statistically significant.

In contrast, muscle mass percentage demonstrated an inverse trend. A significant reduction was observed in the OB group relative to NW (F(2, 28) = 4.595, *p* = 0.019; Figure 1B), indicating a lower proportion of lean tissue among individuals with obesity. The mean muscle mass percentage in the NW group was 33.24% (CI 95% (29.05 to 37.44)); the OW mean was 27.8% (CI 95% (24.54 to 31.06)); the OB mean was 22.08% (CI 95% (19.81 to 24.34)).

### 3.3. Visceral Fat and Metabolic Consumption

**Figure 2:**
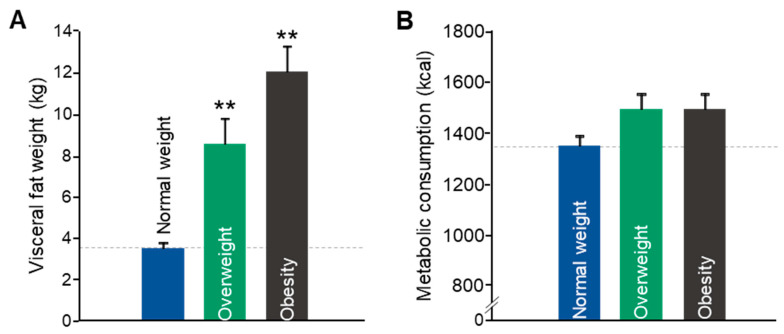
Visceral fat weight (**A**) and metabolic consumption (**B**) in young adults. Data are shown as means ± SEMs. NW (n = 64); OW (n = 25); OB (n = 7). Statistical differences were detected by one-way ANOVA for independent measures followed by Tukey’s b post hoc test (** *p* < 0.0001 vs. rest of the groups).

Visceral fat mass increased progressively across the BMI spectrum, with significantly higher values in both OW and OB groups compared to NW (F(2, 28) = 26.428, *p* < 0.001; Figure 2A). The mean visceral fat weight in the NW group was 3.47 kg (CI 95% (2.98 to 3.97)); the OW mean was 8.5% (CI 95% (5.63 to 11.36)); the OB mean was 12 kg (CI 95% (4.54 to 19.46)).

Metabolic consumption also showed an upward trend in the OW and OB groups (Figure 2B), although the difference did not reach statistical significance (F(2, 28) = 2.667, *p* = 0.087). The mean metabolic consumption in the NW group was 1377.84 kcal (CI 95% (1292.17 to 1463.51)); the OW mean was 1526.38 kcal (CI 95% (1385.63 to 1667.12)); the OB mean was 1528.5 kcal (CI 95% (1248.64 to 1808.37)).

### 3.4. Glucose Tolerance

**Figure 3:**
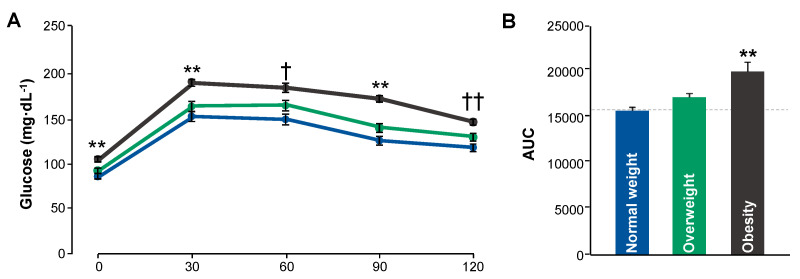
Glucose tolerance profiles (**A**) and corresponding area under the curve (**B**) in young adults stratified by BMI category. Data for each group includes means ± SEMs for each group. NW (n = 64); OW (n = 25); OB (n = 7). Statistical differences were determined using two-way ANOVA for panel A and one-way ANOVA with Tukey’s b post hoc test for panel B (** *p* < 0.001 vs. all groups; †† *p* < 0.001 vs. NW; † *p* < 0.05 vs. NW).

Figure 3A illustrates blood glucose responses over a 120 min period following the administration of a 75 g oral glucose load. The obese (OB) group exhibited significantly higher fasting glucose levels than the normal-weight (NW) and overweight (OW) groups. Post-load glucose levels peaked at 30 min in all groups, with the highest values observed in the OB group. Statistically significant differences were detected at all time points between OB and the other groups.

Although the OW group displayed intermediate glucose levels, these were not statistically different from those in the NW group. By 60 min, glucose concentrations began to decline but remained consistently elevated in the OB group throughout the test period. Two-way ANOVA confirmed significant group-by-time interaction effects.

Figure 3B shows the area under the curve (AUC) for glucose, which increased progressively across BMI categories. The OB group demonstrated the highest AUC, significantly exceeding values in both OW and NW participants (*p* < 0.001 vs. rest of the groups). These differences were confirmed using one-way ANOVA with Tukey’s b post hoc analysis. The mean AUC in the NW group was 15682.5 (CI 95% (14988.15 to 116376.85)); the OW mean was 17135.4 (CI 95% (16,291.32 to 17,979.48)); the OB mean was 19,917.86 (CI 95% (11,7402.11 to 22,433.6)).

Overall, the data suggest that higher body weight is associated with impaired glucose tolerance, leading to prolonged hyperglycemia following glucose ingestion. These findings support the notion that obesity negatively impacts glucose metabolism.

## 4. Discussion

The global rise in overweight and obesity among young adults has become a major public health concern. In our sample of young adults aged 18–25 years, 26.04% were classified as overweight and 7.29% as obese. These prevalence rates were slightly lower than those reported in prior European studies, where overweight and obesity affected approximately 40% and 12–15% of young individuals, respectively [23,24,25].

This lower prevalence may reflect regional differences across Europe. Countries in Northern and Central Europe report higher rates of youth overweight and obesity, particularly where adherence to the Mediterranean diet is weaker [17,23,24]. By contrast, Southern countries such as Spain, Italy, and Greece have shown more favorable anthropometric profiles, although the spread of Western dietary habits is narrowing this gap. Globally, adolescent obesity remains significantly higher in the United States, where estimates exceed 20% [17]. These trends align with forecasts predicting a continuous rise in youth obesity worldwide through 2050 [17,23,24].

Beyond educational attainment, socioeconomic status (SES) is a well-established determinant of obesity risk. Lower SES is linked to poorer access to nutritious food, fewer opportunities for physical activity, and exposure to obesogenic environments [26]. In Spain, García et al. (2025) showed that SES, lifestyle, and regional disparities significantly influence obesity prevalence [27]. Similarly, Tomas-Gallego et al. (2025) reported that students with lower SES had worse adherence to the Mediterranean diet due to financial limitations and limited access to healthy options [28]. These results support the need to incorporate socioeconomic and cultural determinants into early prevention strategies.

For our sample, two protective factors may explain the lower prevalence of obesity. First, the participants were university students, whose higher educational levels (either personal or familial) are associated with reduced obesity risk [29,30,31]. Second, most were from southern Spain, a region with relatively high adherence to the Mediterranean diet, which is known to reduce long-term obesity risk [32].

Our findings confirm that individuals in the obese group had significantly higher total and visceral fat and lower muscle mass compared to those with normal weight. These results were in line with existing evidence suggesting that muscle mass is inversely related to fat accumulation and metabolic dysfunction [33], and that visceral fat is a key predictor of metabolic syndrome [34].

There is an increasing recognition of the interrelated roles of low muscle mass, excess fat, and the development of T2D [35]. Skeletal muscle, beyond its role in locomotion, functions as an endocrine organ that secretes myokines, which regulate systemic metabolism [36]. The disruption of this system due to obesity or sarcopenia may impair insulin sensitivity and contribute to metabolic deterioration [37].

The association between altered body composition and impaired glucose tolerance in our cohort could be explained by disrupted muscle–fat crosstalk. Obesity is associated with altered myokine secretion, ectopic fat accumulation, and low-grade chronic inflammation—all of which impair glucose uptake and insulin action [37,38]. These changes may underline the early metabolic disturbances observed in obese young adults. Targeting these pathways may help delay or prevent T2D onset [39].

In previous studies by our group, body composition was also linked to cognitive function in older adults, with greater muscle mass and lower fat associated with better outcomes [20]. Animal studies have shown that high-fat diets lead to not only metabolic changes but also increased markers of neuronal damage [40]. These findings suggest that obesity-related metabolic dysfunction may affect the brain even in early stages, potentially influencing cognition and behavior through mechanisms involving insulin resistance, inflammation, and disrupted central signaling.

Overweight and obesity are caused by a sustained imbalance between energy intake and expenditure. Although metabolic rate is largely influenced by lean mass, our results suggest that visceral fat contributes significantly to metabolic impairment. The pro-inflammatory profile of visceral adipose tissue—mediated by oxidative stress, lipolytic activity, and immune cell infiltration—plays a central role in insulin resistance [41,42,43]. These processes impair insulin signaling and promote fat accumulation in the liver and muscles [44,45,46].

Our OGTT results revealed that obese individuals had higher post-load glucose levels and greater AUCs, reflecting reduced glucose tolerance. While these findings mirror those in adults [47], their presence in a population aged 18–25 years is concerning and highlights the early onset of metabolic dysfunction [48,49]. The mechanisms are likely multi-factorial and include visceral fat, muscle loss, and changes in endocrine and inflammatory signaling [50].

These alterations may be the early signs of a progressive metabolic decline. The persistence of visceral adiposity and insulin resistance from early adulthood has been linked to future risk of T2D, cardiovascular disease, and cognitive decline [20,51]. As shown by our group and others, sarcopenic obesity correlates with poor physical and cognitive outcomes in older adults [39]. Addressing these patterns early may be essential to reduce the long-term burden of chronic disease and functional decline.

### 4.1. Implications for Prevention, Clinical Practice, and Future Research

Our findings suggest several clinical and public health implications. First, simple tools such as bioelectrical impedance could be integrated into routine assessments in youth to detect early metabolic risk. These methods are cost-effective and suitable for large-scale screening.

Second, digital tools—such as mobile applications for lifestyle tracking or glucose monitoring—could support personalized interventions in young populations. Third, the adoption of a life-course approach focused on early detection and tailored prevention strategies during the transition to adulthood is strongly warranted.

### 4.2. Limitations

This study had several limitations. First, the cross-sectional design prevents causal inferences. Second, although an ANOVA could have offered a more integrative multivariate analysis of glucose metabolism and body composition, it was not conducted due to collinearity among variables and sample size limitations. Third, although a sex-stratified analysis was performed, the imbalance between male and female participants—particularly the smaller male group—limited statistical power and reduces the generalizability of the findings to women. The small number of participants in the obese group (n = 7) limits the statistical robustness and precision of group comparisons, and the results should be interpreted with caution. Additionally, participants were recruited from a single region and may not reflect the full sociodemographic diversity of the young adults. Future studies should explore sex-specific differences, longitudinal progression, and the effectiveness of multicomponent interventions, including dietary, physical-activity, and technology-based strategies aimed at preventing the onset of T2D in this vulnerable age group.

## 5. Conclusions

Taken together, our findings demonstrate that young adults with obesity already present significant alterations in body composition, including in increased total and visceral adiposity, reduced muscle mass, and impaired glucose tolerance. These results emphasize that obesity in early adulthood is not merely a matter of excess body weight but is associated with meaningful metabolic disturbances that may predispose individuals to chronic diseases in both the short and long term.

Preventive and therapeutic strategies targeting improvements in body composition and metabolic function at an early stage are therefore essential to mitigate the future burden of metabolic disease and its associated complications.

## Figures and Tables

**Table 1:** Cohorts, body height, body weight, and basal mass index ± each SEM in the different groups.

	Cohort (%)	Height (m)	Weight (kg)	BMI
Normal weight	66.67 (n = 64)	1.67 ± 0.01	60.92 ± 1.04	21.76 ± 0.21
Overweight	26.04 (n = 25)	1.65 ± 0.01	71.56 ± 1.59 **	26.09 ± 0.2 **
Obesity	7.29 (n = 7)	1.63 ± 0.02	86.57 ± 4.56 **	32.59 ± 1.35 **

** *p* value < 0.001 vs. rest of the groups.

## Data Availability

Data is contained within the article.

## Abbreviations

The following abbreviations have been used in this manuscript:

T2D	Type 2 Diabetes
BMI	Body Mass Index
NW	Normal Weight
OW	Overweight
OB	Obesity
AUC	Area Under Curve
SES	Socioeconomic Status
